# Retrospective analysis of protein kinase C-beta (PKC-β) expression in lymphoid malignancies and its association with survival in diffuse large B-cell lymphomas

**DOI:** 10.1186/1745-6150-2-8

**Published:** 2007-02-21

**Authors:** Shuyu Li, Mark Phong, Michael Lahn, Leslie Brail, Susan Sutton, Boris K Lin, Donald Thornton, Birong Liao

**Affiliations:** 1 Integrative Biology Informatics, Eli Lilly and Company, Lilly Corporate Center, Indianapolis, IN 46285, USA; 2 Medical Oncology, Lilly Research Laboratories, Eli Lilly and Company, Bad Homburg, Germany; 3 Medical Oncology, Lilly Research Laboratories, Eli Lilly and Company, Indianapolis, IN 46285, USA

## Abstract

**Background:**

Both mechanistic features and recent correlative findings suggest a potential role for protein kinase C-beta (PKC-β) in tumor pathogenesis, particularly in B-cell malignancies. To evaluate the role of this gene in lymphoid malignancies, we analyzed global gene expression data to quantify PKC-β expression across diagnostic groups and, when possible, determined correlations between PKC-β expression and survival.

**Results:**

Our analysis showed that the level of PKC-β expression was highest in chronic lymphocytic leukemia and follicular lymphoma. Within diffuse large-B cell lymphoma (DLBCL), PKC-β expression was significantly higher in activated B-cell- like subtype than germinal center B-cell- like subtype (*P *< 0.0001). Elevated PKC-β appeared to be associated with worse survival in both of these subtypes. When analyzed within clinically defined risk groups established by the International Prognostic Index (IPI), PKC-β expression was lowest in patients with low IPI scores (0–1). Within intermediate- and high-risk IPI groups, elevated PKC-β expression was associated with worse survival, suggesting that PKC-β may expand the prognostic value of the IPI. Results of global gene expression analyses of DLBCL samples corroborate previous observations that anti-apoptosis, cell proliferation, and B-cell proliferation signaling pathways are functionally related to PKC-β.

**Conclusion:**

We present a first detailed pharmacogenomics report comparing PKC-β mRNA expression across different lymphoid malignancies and evaluating it as an outcome predictor. Our findings suggest that DLBCL patients with elevated PKC-β have a worse prognosis, indicating that further evaluation of PKC-β as a chemotherapeutic target for lymphoid malignancies is warranted.

**Reviewers:**

This article was reviewed by Dr. Pierre Pontarotti, Dr. Kateryna Makova, and Dr. Matthew Coleman (nominated by Dr. Sandrine Dudoit).

## Background

The protein kinase C (PKC) family of serine/threonine kinases has an important role in biological processes related to signal transduction, cell proliferation, and apoptosis. The PKC-β 1 and 2 isoforms? have been implicated in several biological pathways and could serve as targets for cancer chemotherapy [1,2].

Protein kinase C-beta (PKC-β) is a regulator of the vascular endothelial growth factor (VEGF) signal cascade and angiogenesis [3-7]. Xia et al demonstrated that PKC inhibitors could block VEGF-induced PKC activation and endothelial cell proliferation [7]. Additionally, VEGF-mediated mitogenic effects were blocked by PKC-β inhibitor, LY333531, but not by antisense oligonucleotides to PKC-α [7].

PKC-β is a key component of the B-cell receptor (BCR) signaling pathway and is clearly involved in the normal function of B-cells. PKC-β knockout mice have impaired humoral and B-cell proliferative responses [8]. BCR stimulation of PKC-β-deficient B cells failed to up-regulate the anti-apoptotic protein Bcl-xl, and quenched cell survival signals [9]. Likewise, in intact B cells, PKC-β activation downstream of BCR antigen binding results in immature B-cell proliferation and survival [10]. PKC-β inhibitors reduced the cell viability of two diffuse large B-cell lymphoma (DLBCL) cell lines, SUDHL6 and OCILY10, that have over-expressed PKC-β [9]. The PKC-β inhibitor enzastaurin has shown activity in xenograft mouse models of diffuse large cell lymphoma [11].

These findings suggest that PKC-β likely performs a pivotal role in the pathogenesis of malignant B cells. Shipp et al used gene expression profiling and supervised learning methods with newly diagnosed DLBCL samples to identify genes that were differentially expressed in fatal/refractory versus cured subgroups [12]. Their analysis produced a multi-gene outcome prediction model that provided unique information not represented in the existing clinical prognostic model. From this study it was determined that PKC-β was one of the most prominently over-expressed genes in fatal/refractory DLBCL patients (n = 58; P = 0.03). Shipp et al also reported that PKC-β protein expression was positively associated with transcript abundance and closely associated with clinical outcome in a pilot series of DLBCL patients (n = 21; P = 0.03).

The availability of microarray gene expression databases in the public domain provides a potentially valuable resource for the identification of critical pathways in cancer treatment. In this retrospective analysis, we examined microarray gene expression databases to determine if PKC-β expression was elevated in various types of lymphoid malignancies. We also examined whether elevated PKC-β expression was associated with worse survival in DLBCL and its subtypes. We evaluated PKC-β to determine whether it could predict survival within the clinically defined risk groups established by the International Prognostic Index (IPI) [13]. To validate the biological relevance of these findings, we evaluated other genes represented on the arrays to identify gene pathways that might be coordinately regulated with PKC-β.

## Results

### PKC-β expression in lymphoid malignancies

**Figure 1:**
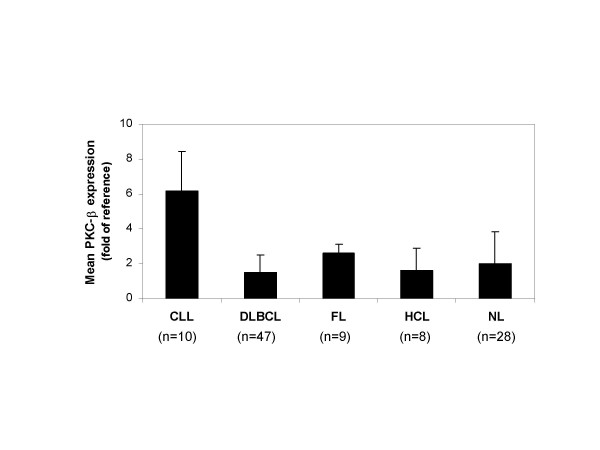
**PKC-β expression in lymphoma subtypes and normal tissues**. Mean PKC-β (isoform 2) gene expression in CLL, DLBCL, FL, hematopoetic cell lines, and normal lymphoid (NL) tissues. Vertical error bars represent standard deviations for each category.

Lymphoma is the fifth most common cancer in the United States and represents over forty subtypes of cancers arising within the lymphatic system. Lymphomas are classified into several large groups by cell type: the B cell tumors, the T cell and natural killer cell tumors, Hodgkin lymphoma, and other minor groups. The most prevalent subtypes include DLBCL, chronic lymphocytic leukemia (CLL), follicular lymphoma (FL), and Mantel cell lymphoma (MCL). Our analysis of PKC-β gene expression across lymphoma subtypes [14] revealed that PKC-β expression was highest in CLL, notably 3-fold greater than in normal lymphoid (NL) samples (CLL vs NL, *P *< 0.0001; Fig 1). Follicular lymphoma cases exhibited higher PKC-β expression than DLBCL cases (*P *< 0.0001). Analysis of a second dataset derived from Affymetrix arrays [12] showed a similar trend (data not shown) with mean PKC-β expression significantly elevated in FL relative to DLBCL samples (*P *= 0.018).

### PKC-β as an outcome predictor in DLBCL

**Figure 2:**
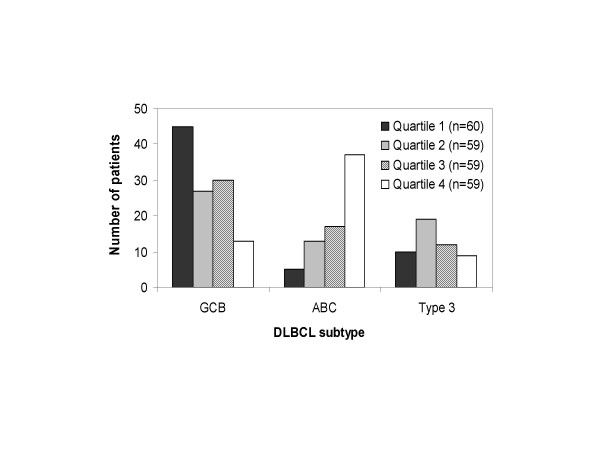
**The distribution of patients in each PKC-β-defined quartile among DLBCL subtypes**. DLBCL subtypes included GCB, ABC, and type 3 as defined by Rosenwald et al (2002). Samples were ordered according to relative expression of PKC-β and assigned to a quartile such that patients with the highest PKC-β expression were represented in quartile 4.

To determine the relationship between PKC-β expression and survival for DLBCL patients, we analyzed three microarray datasets (Table 1). In the Rosenwald et al dataset [15], patients with high PKC-β expression (quartile 4) were disproportionately represented among the three gene expression DLBCL subtypes: germinal center B-cell- like (GCB), activated B-cell- like (ABC), and type 3 (Fig 2). Seventy-five percent (45/60) of the lowest PKC-β-expressing patients (quartile 1) belonged to the GCB subtype (DLBCL subtype with the best long-term prognosis). In contrast, 63% (37/59) of the highest PKC-β-expressing patients (quartile 4) belonged to the ABC subtype, which is characterized by a lower 5-year survival rate. Lymphomas classified as ABC had significantly higher mean PKC-β expression than either type 3 (43% higher, *P *= 0.0011) or GCB (76% higher, *P *< 0.0001).

**Table 1:** Gene expression datasets included in this study

**Reference**	**Cancer type**	**Array platform**	**Sample size**	**URL for data downloading**
Alizadeh et al (2000)	DLBCL, CLL, FL	cDNA (Lymphochip)	102	
Shipp et al (2002)	DLBCL, FL	Affymetrix (HU6800)	77	
Rosenwald et al (2002)	DLBCL	cDNA (Lymphochip)	240	

**Figure 3:**
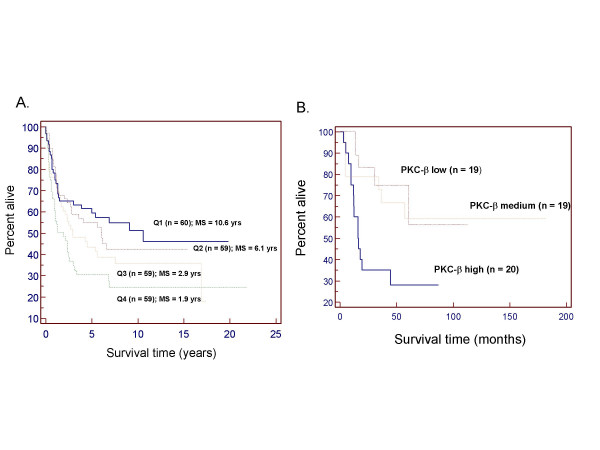
**Correlation between PKC-β gene expression and patient survival in DLBCL**. DLBCL patients in two datasets (A: Rosenwald et al, 237 samples; B: Shipp et al, 58 samples) were separated into four and three groups with an equal number of samples in each group, respectively, based on PKC-β gene expression. Higher quartiles represent groups with higher PKC-β expression. Kaplan-Meier survival curves are shown for each group. Median survival (MS) times are shown when sufficient data were available for each group.

Kaplan-Meier estimates [16] of 237 DLBCL patients showed a negative association between PKC-β gene expression and median survival time (Fig 3A; *P *= 0.0129). Patients with very high PKC-β expression (quartile 4) had a median survival time of 1.9 years, whereas patients with low PKC-β expression (quartile 1) had a median survival time of 10.6 years. Our analysis of expression data for 58 DLBCL samples from Shipp et al [12] showed that patients with high PKC-β expression had shorter survival than patients with low or medium PKC-β expression (Fig 3B; *P *= 0.0126). The association between high PKC-β expression and poor survival indicated in Kaplan-Meier analysis is also confirmed by an alternative approach using the Cox Proportional-Hazards Regression model (data not shown). Applying similar analyses to the Alizadeh et al dataset [14] showed no statistically significant association between high PKC-β expression and overall survival, possibly due to a relatively small sample size.

**Figure 4:**
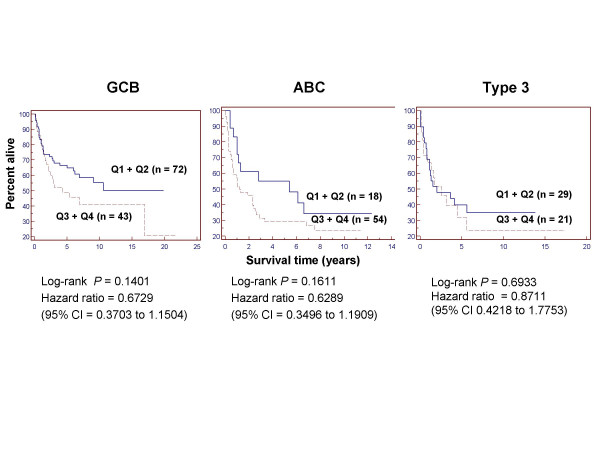
**Kaplan-Meier plots of overall survival for GCB, ABC, and type 3 DLBCL**. Subtypes were stratified according PKC-β expression. Quartile 1 was combined with quartile 2 and quartile 3 was combined with quartile 4 to allow for more robust comparisons. Higher quartiles represent groups with higher PKC-β expression.

We further analyzed data from Rosenwald et al [15] to determine if PKC-β-defined subgroups differed with respect to median survival times within the DLBCL subtypes ABC, GCB, and type 3 (Fig 4). To establish larger subgroups for more robust comparisons, we combined quartiles 1 and 2 and then combined quartiles 3 and 4. Observed differences in survival between these PKC-β defined subgroups were not statistically significant but trends were consistent with previous analyses.

### Comparison of a PKC-β-defined outcome predictor and the IPI

**Figure 5:**
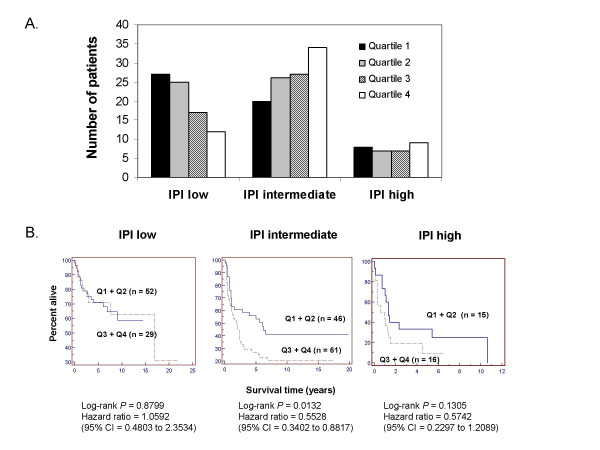
**Relationship between PKC-β gene expression as an outcome predictor and the international prognostic index (IPI)**. The IPI classifies patients into 3 risk groups: low-risk, defined by an IPI score of 0–1; intermediate-risk, defined by an IPI score of 2–3; and high-risk, defined by an IPI score of 4–5. IPI scores were unknown for 18 patients. A. Number of patients represented in each PKC-β defined quartile for the IPI low-, intermediate-, and high-risk subgroups. B. Kaplan-Meier plots of overall survival for IPI low-, intermediate-, and high-risk subgroups, stratified according to the quartile of PKC-β expression. Quartile 1 was combined with quartile 2 and quartile 3 was combined with quartile 4 to allow for more robust comparisons. Higher quartiles represent groups with higher PKC-β expression.

Each of the PKC-β defined quartiles were fairly evenly represented in each of the three IPI risk groups for the Rosenwald dataset [15] (low, intermediate, and high) (Fig 5A). Patients with high PKC-β expression (quartiles 3 and 4) were not overrepresented in the IPI high-risk group. In fact, 57.5% of these patients (61 of 106 patients in quartiles 3 + 4) were categorized as IPI intermediate-risk. PKC-β expression differed significantly across IPI risk groups (ANOVA *P *= 0.008). PKC-β expression was significantly higher in IPI intermediate-risk patients than in IPI low-risk patients (1.4-fold difference; *P *= 0.0005). Patients in IPI intermediate- and high-risk subgroups who also had high PKC-β expression (quartiles 3 + 4) showed worse survival (Fig 5B). In the intermediate-risk group, patients with high PKC-β expression had significantly shorter median survival times than patients with lower PKC-β expression (quartile 3 + 4 = 1.9 years vs quartiles 1 + 2 = 6 years, *P *= 0.013).

### Genes differentially expressed in PKC-β-defined subgroups

**Figure 6:**
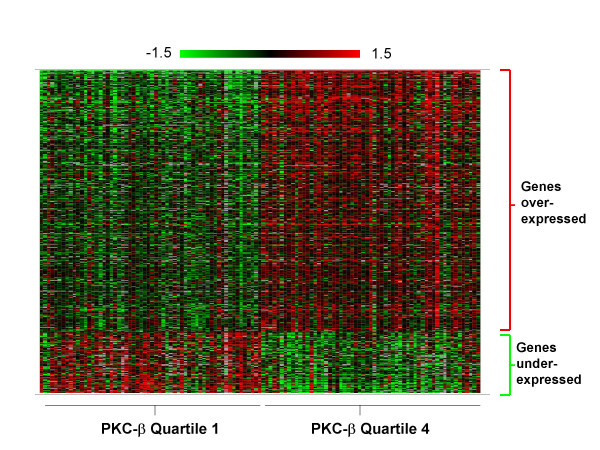
**Genes differentially expressed in high versus low PKC-β expressing DLBCL patients (quartile 4 vs quartile 1)**. Gene expression level is represented by log transformed ratios of hybridization signal from experimental samples versus the reference sample. Expression is shown in colors, with red representing high expression and green representing low expression. 820 cDNA clones were differentially expressed (FDR < 0.01 and missing data points < 40) between the PKC-β defined quartile 4 and 1. 81% of these genes were over-expressed in the highest PKC-β subgroup (quartile 4).

We examined gene expression data for all genes on the cDNA arrays first analyzed by Rosenwald et al [15] to further validate the biologic relevance of PKC-β as a potential prognostic marker for patients with DLBCL. We identified 820 cDNA clones that were differentially expressed between the highest (quartile 4) and the lowest (quartile 1) PKC-β subgroups. Eighty-one percent (664/820) of these clones showed higher expression in quartile 4 than quartile 1, suggesting that they may be functionally related to PKC-β (Fig 6, Table 3). These over-expressed clones were mapped to 345 unique UniGene clusters, with approximately 60% having Gene Ontology annotations that can be linked to related biological processes and pathways. Many of these genes are represented in anti-apoptosis, cell proliferation, B-cell signaling, cell-cycle control, and DNA repair pathways, further confirming the link between PKC-β activation and tumor growth (Table 3, and Additional file 1). Many genes involved in replication, transcription, and translation are also up-regulated, possibly reflecting the high rate of cell proliferation in these patients.

**Table 3:** Categories of genes over-expressed in the highest PKC-β expressing (quartile 4) DLBCL patients

Anti-apoptosis
Signaling in B-cell proliferation, differentiation
Cancer development
Cell proliferation/cell-cycle control
DNA repair
DNA, RNA, protein synthesis

## Discussion

When validating any gene or gene product as a viable target for cancer chemotherapy, it is important to move beyond in vitro models and understand how the target gene expression affects disease progression and how it varies in patient populations. Global gene expression analysis with DNA microarray technology has proven to be a useful tool for target identification and validation in both the preclinical and clinical settings. Gene expression analysis is currently used for diagnosis, prognosis, and therapeutic decision-making in a variety of cancers. Retrospective analysis of public microarray data associated with clinical studies in primary cancers allows us to evaluate potential targets, and validate their prognostic significance across larger patient populations and more tumor types. Analyzing microarray gene expression data in publicly available database is a valuable approach to generate novel findings using the existing data. Here, we present the first detailed pharmacogenomics report comparing PKC-β mRNA expression across different lymphoid malignancies and evaluating it as an outcome predictor.

Our analysis of PKC-β across different diagnostic categories revealed significantly higher expression in CLL patients as compared to normal lymphoid tissues (Fig 1). When the expression of a potential drug target is higher in tumor cells than in normal cells, there is an implication that the tumor cell requires that target for its survival; thus, it follows that blocking the target would impede cancer cell proliferation. Elevated expression of PKC-β in FL relative to DLBCL suggests that PKC-β could be important to the pathogenesis of FL. A correlation between PKC-β expression and survival in CLL and FL patients would further establish the importance of PKC-β as a viable target for cancer chemotherapy. Although this line of investigation is of considerable interest, these analyses were not done due to the lack of clinical information accompanying gene expression data.

An alternative approach to target validation is to discover genes (or gene products) as potential drug targets that are elevated in a subset of cancer patients with poor clinical outcomes. This may be the case with PKC-β in DLBCL. Each of the two major gene expression profiling studies that characterized DLBCL samples yielded a multigene predictive model (17 genes in Rosenwald et al and 13 genes in Shipp et al), but only the 13-gene model presented by Shipp et al included PKC-β as a negative predictor for survival [12,15]. In fact, there was no overlap between genes represented in the two models. This discrepancy could, at least in part, be attributed to different patient populations, different array platforms, and/or different analysis algorithms.

Our analysis of PKC-β expression in the Rosenwald dataset [15] yielded results consistent with the observations of Shipp et al [12]. High PKC-β expression was associated with shorter overall survival time. Our new analysis of the Shipp dataset showed similar trends. The outcome prediction model applied in Rosenwald et al [15] clustered DLBCL patients into three subtypes: GCB, ABC, and type 3, on the basis of cell-of-origin gene expression patterns. Our analysis showed significantly higher PKC-β in the ABC as compared to the GCB subtype. The biological significance of this finding was highlighted by our observation that within the ABC subtype, high PKC-β-expressing patients (quartile 3 + 4) appeared to have shorter median survival times (Fig 4), although the results were not statistically significant. PKC-β expression appears to provide unique prognostic information that is not reflected in the cell-of-origin gene signature model for DLBCL. Analysis of survival curves for PKC-β stratified subgroups within each IPI risk group revealed equivocal findings (Fig 5B). For the IPI intermediate-risk group, elevated PKC-β expression was significantly associated with worse survival, and it appears that this biomarker may provide unique prognostic information not represented in the clinical prognostic model. A similar trend but statistically insignificant was evident with IPI high-risk patients, but there was no association for the IPI low-risk group. This lack of significance may be a sample size limitation, but other factors may also be important. Perhaps additional genetic biomarkers or clinical measures could be combined with PKC-β to improve predictive power and to better characterize the relative risks for patients in the IPI low- or high-risk groups.

Our observations with DLBCL patients reinforce previous findings that PKC-β gene expression likely contributes to the pathological processes associated with DLBCL. These findings are complimented by a number of studies that used tissue arrays to demonstrate an association between increased PKC-β protein expression and shorter 5-year disease-free survival [12,17,18]. Similarly, Hans et al [19] applied multivariate analysis to tissue array data and showed that patients who expressed either PKC-β or cyclin D2 had a 5-year overall survival of 30% compared with 52% for patients who were negative for both markers.

Gene annotation and pathway analyses presented in the current report illustrated that genes in anti-apoptosis, cell proliferation, B-cell proliferation/differentiation, cell-cycle control, and DNA repair pathways are over-expressed in PKC-β-defined quartile 4 (Table 3, and Additional file 1). We observed elevated expression of ribosomal protein S6 kinase, phosphoinositide-3-kinase, and transforming growth factor β in high PKC-β expressors, supporting the notion of cross-talk among these signaling pathways [20,21]. Up-regulation of these pathways inhibits B-cell apoptosis and facilitates cell proliferation. These results confirm that PKC-β is clinically important to BCR signaling. Genes identified in this analysis may, after further research, prove to be important as functional biomarkers of PKC-β inhibition. Additionally, these findings may direct us to other signaling pathways and specific genes therein that are potential targets for cancer chemotherapy.

## Conclusion

Data mining, as presented here, provides an integrated approach to target assessment and validation for drug discovery and development. Our analysis showed that for DLBCL patients, elevated PKC-β expression was associated with worse survival. Previously defined DLBCL subgroups characterized by decreased survival (e.g. ABC and type 3 DLBCL) also had elevated PKC-β expression. Additional studies aimed at validating this target in DLBCL patients are warranted and should include patients that would now typically receive rituximab as part of their therapeutic regimen. PKC-β is also significantly elevated in CLL and FL. Information is still needed to determine whether the trend observed in DLBCL between high PKC-β expression and poor survival is mirrored in these cancers. Taken together, these findings further strengthen previous assertions that this gene could likely be an important target for cancer chemotherapy.

## Methods

For all analyses, we used gene expression data and corresponding clinical information available on the internet (Table 1).

### Analyses of PKC-β expression in lymphoid malignancies

We assessed PKC-β gene expression in different lymphoma subtypes by analyzing data from Alizadeh et al of 102 samples of DLBCL, CLL, FL, hematopoetic cell lines, and subsets of NL tissues [14]. This gene expression profiling dataset from Lymphochip cDNA arrays, included genes preferentially expressed in lymphocytes or genes with known or possible roles in lymphocyte differentiation and proliferation under normal or malignant conditions. We focused on data representing PKC-β isoform 2 here, but analyses of PKC-β isoform 1 clones yielded similar results.

Using a second dataset, first reported by Shipp et al, we assessed PKC-β expression in 58 DLBCL and 19 FL samples [12]. Affymetrix HU6800 was the microarray platform (Affymetrix, Santa Clara, CA). We analyzed results from two probesets representing PKC-β: X06318_at (PKC-β isoform 1) and X07109_at (PKC-β isoform 2). Expression data reported as average difference were generated by Affymetrix MAS 4.0. Expression data from X06318_at and X07109_at were highly correlated (Pearson's correlation coefficient R = 0.82); therefore, only data from one probeset, X07109_at, are presented.

### Analyses of PKC-β as an outcome predictor in DLBCL and DLBCL subtypes

To examine the relationship between PKC-β expression and survival in DLBCL patients, we analyzed three microarray datasets (Table 1). All DLBCL patients evaluated received anthracycline-based chemotherapy, which usually consisted of cyclophosphamide, doxorubicin, vincristine, and prednisone (CHOP).

The most extensive microarray dataset was generated by Rosenwald et al [15] as part of the Lymphoma/Leukemia Molecular Profiling Project (LLMPP). Gene expression analysis was performed on biopsy samples from 240 DLBCL patients using the Lymphochip. A total of six clones were annotated as PKC-β (Table 2). After analyzing the sequence of these six clones, we chose 19235 (GenBank:AA243358, PKC-β isoform 2) and 28417 (GenBank:AA479102, PKC-β isoform 1) corresponding to the 3'-UTR of PKC-β for further analysis. Nucleic acid sequences in the 3'-UTR are highly specific and cross hybridization of 3'-UTR to other genes in the same gene family on microarrays is minimal. Clones not mapped to the 3'-UTR of the PKC-β gene (34136, 32251, and 24623) and those missing a significant amount of data in 240 DLBCL samples (15858) were omitted from further analysis. Gene expression data for 19235 and 28417 were strongly correlated (Pearson's correlation coefficient 0.84), and data from clone 19235 are presented. For clone 19235, 237 data points were available from 240 patients.

**Table 2:** Clones on Lymphochip cDNA arrays representing PKC-β

**Clone ID**	**GenBank accession**	**Sequence mapping region**	Available data points from 240 DLBCL samples*
34136	AI540068	5'-UTR + exon1	229
15858	AA479102	3'-UTR	165
28417	AA479102	3'-UTR	236
32251	AA262174	Intron	225
24623	AA830034	Not mapped to PKC-β	212
19235	AA243358	3'-UTR	237

* Rosenwald *et al *(2002).

When analyzing the data from Rosenwald et al [15], we ordered the lymphoma cases according to relative expression of PKC-β and assigned each to a quartile in ascending order so that patients with the highest PKC-β expression were represented in quartile 4. Quartile 1 included 60 patient samples and quartiles 2–4 each included 59 patient samples. We analyzed the association between PKC-β expression and patient survival for the entire DLBCL group and compared PKC-β gene expression across DLBCL subtypes (GCB, ABC, and type 3) which were previously defined in the multi-gene prediction model [15].

As part of their original analysis, Rosenwald et al [15] showed that the gene signature scores and IPI scores [13] were independent predictors of survival. We compared PKC-β gene expression across these IPI groups and analyzed the association between PKC-β gene expression and survival within each group.

Using DLBCL gene expression data from Shipp et al [12] (58 samples), we assigned lymphoma samples to one of three groups characterized by high, medium, and low PKC-β expression (subgroup sizes were 20, 19, and 19, respectively) and evaluated the association between PKC-β expression and survival. We conducted similar analyses using data from Alizadeh et al [14] of 35 DLBCL samples (classified as either high or low PKC-β expressors).

### Analyses of genes differentially expressed in PKC-β-defined subgroups

To determine which genes might be coordinately regulated with PKC-β, we evaluated other genes represented on the arrays and identified clones that varied significantly between the two most extreme PKC-β defined subgroups (highest: quartile 4 vs lowest: quartile 1).

### Statistical methods

Two-tailed t-tests were used for within-dataset comparisons of PKC-β gene expression in different lymphoma subtypes. Fisher's exact tests (using JMP 5.1) were used to compare patient counts across DLBCL subtypes (GCB, ABC, type 3), and across IPI risk groups. One way ANOVA and Student's t-tests with the Bonferroni multiple comparison adjustment were used for pair-wise comparisons of PKC-β gene expression between DLBCL subtypes and IPI risk groups. The Kaplan-Meier method [16] was used to estimate median survival time and the log-rank test was used to compare survival rates among DLBCL subtypes and PKC-β-defined subgroups. Time-to-event analyses were performed in MedCalc (MedCalc Software, Mariakerke, Belgium).

Using a two-sided Student's t-test, we identified genes differentially expressed between PKC-β-defined subgroups (highest vs lowest quartile). To account for multiplicity, the Benjamini and Hochberg [22] false discovery rate control (FDR) adjustment was applied as FDR = (*P *× *n*)/*i*, in which *P *is the *P *value from the t-test, *n *is the total number of genes, and *i *is the sorted rank of the *P *value. Using criteria FDR < 0.01 and missing data points < 40, we identified cDNA clones that were differentially expressed between the highest and the lowest PKC-β subgroups.

## Authors' contributions

DT and BL conceived of the study. SL, MP and BL carried out data analyses. SL, and BL drafted manuscript. ML, LB, BKL and DT contributed to clinical interpretation of results and toward revising the manuscript. SS contributed to discussions about statistical analysis, interpretation of results and revision of the manuscript. All authors read and approved the final manuscript.

## Reviewers' comments

### Reviewer's report 1

Dr. Pierre Pontarotti, Université d'Aix Marseille, Marseille, France

The authors perform a retrospective analysis showing that Diffuse Large B cell lymphoma patients with elevated expression of protein Kinase C-Beta gene have worse prognosis.

This is an important confirmative result that should be published.

I like very much the idea of performing retrospective analysis using available data.

I recommend the authors to make a brief comment on the state of the art on such approaches and especially in the case of microarray gene expression databases. I think that this kind of approach will be more and more used in the future.

#### Authors' response

*We appreciate the comments. Particularly, we agree with the reviewer that retrospective analysis of available microarray gene expression data is a "state of art" approach in biomedical research to generate novel findings by using the existing data. As recommended by the reviewer, we added several sentences in the 1^st ^paragraph of the discussion to emphasize this point*.

### Reviewer's report 2

Dr. Kateryna Makova, Pennsylvania State University, Pennsylvania, United States

In this paper the authors show that increased PKC-b expression in some types of cancers can be correlated with a shorter survival time after treatment.

Major Criticisms

General:

Authors should highlight the novelty of this study better.

#### Author's response

*We followed the reviewer's advice and highlighted the novelty in both abstract and the discussion section. For example, we added the statement "We present a first detailed pharmacogenomics report comparing PKC-β mRNA expression across different lymphoid malignancies and evaluating it as an outcome predictor" in the abstract*.

Results:

1. In general this section could be expanded, in particular section on gene expression analysis that will be of interest to genomicists.

#### Author's response

*We agree with reviewer's opinion. Please see below our responses to each of reviewer's specific suggestions*.

2. Readers may be unfamiliar with the lymphoma subtypes, and so a description of each would be beneficial.

#### Author's response

*We followed the reviewer's advice and added a paragraph at the beginning of the result section to briefly describe the lymphoma subtypes*.

3. When comparing differentially expressed clones between quartiles 1 and 4 of PKC-b expression, it would be useful to compare both quartiles to healthy lymphatic tissues as a control.

#### Author's response

*We completely agree with reviewer's opinion. However, the Rosenwald dataset that we analyzed did not include healthy lymphatic tissues as a control. Therefore, we could not compare PKC-β expression in DLBCL with that of healthy control tissues*.

4. There are now protein-protein interaction studies that can be included in the study to identify the protein partners of the expressed mRNA.

#### Author's response

*We agree with the reviewer that protein-protein interaction data could provide more insights into the role of PKC-β in DLBCL. However, the main goal of the current study was to evaluate PKC-β as a target of B lymphocyte malignancies by analyzing its expression across subtypes and correlation between expression and survival, and our results have clearly indicated that PKC-β could be an important target for B cell lymphoma chemotherapy. Protein-protein interaction would exceed the scope of this study*.

5. What genes are under-expressed in the quartiles, compared to each other, and compared to healthy tissues?

#### Author's response

*We followed the reviewer's suggestion and analyzed genes under-expressed in quartile 4(high PKC-β expressors). Unlike those over-expressed genes, we did not observe specific pathways with significantly under-expressed genes correlated with high PKC-β expression*.

Discussion:

1. It is a little confusing that the comparison of PKC-b expression across different kinds of cancers is included at the beginning of the Discussion section. This seems to justify further studies of PKC-b in CLL and FL cancers because it was shown to have potential predictor roles in DLBCL, even though PKC-b expression is much lower in DLBCL cancers than either CLL or FL. Maybe this paragraph should be moved to the end of the section, to maintain the focus of the paper on PKC-b expression in DLBCL cancers.

#### Author's response

*We agree with the reviewer regarding further studies of PKC-β expression in CLL and FL. However, in the dataset we analyzed, survival data was not available for CLL and FL for more detailed analysis of PKC-β in these two subtypes, as indicated in the 2^nd ^paragraph of the discussion section*.

2. The authors claim that, "A similar association was evident with the IPI high-risk patients, but there was no discernable trend for the IPI low-risk group." This seems like a strong statement to make. From 5B, it seems that survival rates are the same for all expression levels in the IPI low-risk group, not that there is no discernable trend. Also, it is difficult to assert that the trend is the same in both the IPI intermediate and IPI high risk group, especially with the small sample sizes and insignificant p-value.

#### Author's response

*We agree with the reviewer and modified the wording in the sentence to indicate that the association between PKC-β expression and survival was not statistically significant in the high IPI group and there is completely lack of association in the low IPI group*.

3. The authors claim that, "These results confirm that PKC-b is clinically important to BCR signaling." This is not necessarily true, unless one can show that it is the upregulation of PKC-b that is causing the other factors to be up-regulated, and not that PKC-b is just another byproduct of some other up-regulation.

#### Author's response

*Our statement is based on previous studies, for example, please refer to reference 9 for the role of PKC-β in BCR signaling*.

4. To exclude the possibility of cross-hybridizations amplifying PKC-b expression signals the authors could check for the uniqueness of the probes to PKC-b on all of the arrays (both cDNA and affymetrix).

#### Author's response

*We agree with the reviewer. The issue has been addressed by detailed discussion of probe sequences in *Table 2*and the method section*.

Minor Criticisms

Abstract:

1. We suggest that the sentence be changed to reflect the gene expression from the microarrays – "Our analysis showed that the level of PKC-b expression was..."

2. We suggest that the sentence specify the expression of PKC-b – "...lymphoma (DLBCL), the level of PKC-b expression was significantly..."

3. Given that the p-value is significant, it doesn't seem necessary to also include it in a parenthetical reference.

Background:

1. To clarify the two PKC-b isoforms, it would be useful to change the format to something similar to – "The PKC-b1 and -b2 isoforms..."

2. There is no citation for "Additionally VEGF-mediated mitogenic effects were blocked by PKC-b inhibitor LY333531, but not by antisense oligonucleotides to PKC-a."

Results:

1. To clarify that the subtypes are being listed, we suggest using a colon after, "... among the three gene expression DLBCL subtypes: germinal ..."

2. On figure 3A, it is difficult to see some of the lines, so we suggest to bolden the Q3 lines.

3. On figure 3B, we suggest to make the PKC-b medium line bolder.

4. On figure 3B, we suggest to change the x-axis to years instead of months, to be consistent with the other graphs. Also, for consistency, it might be nice to see it divided into quartiles, instead of thirds.

5. On figure 5B, in all three plots, it is difficult to distinguish the Q3+Q4 line in the on-line version and would be much easier to read if this line were darker

Discussion:

1. In the first sentence"know" to "understand."

2. There is no citation for, "Global gene expression analysis with DNA microarray technology has proven to be a useful tool for target identification and validation in both the preclinical and clinical settings."

3. There is no citation for, "Gene expression analysis is currently used for diagnosis, prognosis and therapeutic decision-making in a variety of cancers."

4. Change "PKC-B" to "PKC-b"

5. Change from "drugable genes" to "genes that are potential drug targets."

6. Change to a colon after, "...clustered DLBCL patients into three subtypes: GCB, ABC..."

7. There is no reference to figure 2 when it is mentioned in, "Our analysis showed significantly higher PKC-b in the ABC..."

8. The reference should be to Table 3, not Table 2, after, "Gene annotation and pathway analyses presented in the current report..."

#### Author's response

*We have modified the manuscript to address every point in reviewer's comments, except for the suggestion to change the boldness of lines in *Figure 3B*and *5B. *Since the figures were generated using the software MedCalc (see the Method's section), we could not modified each lines. However, since the lines are in color, we feel the readers should be able to distinguish different survival curves that were represented by different colors*.

### Reviewer's report 3

Dr. Matthew Coleman, Lawrence Livermore National Labs, Livermore, CA 94550. Nominated by Dr. Sandrine Dudoit.

Reject until major revisions are made. Scientific quality of the work is high and should be of general interest once major revisions are made. The paper would have been strengthened by multiple statistical testing of the hypotheses (see comments below). The tables and figures should be restricted to those that are critical. All other figures/tables should be moved to a supplemental data section.

General Comments for the Author:

The authors take a statistical and bioinformatics approach to rexamine the role for protein kinase C-beta (PKC-b) in tumor pathogenesis related to B-cell malignancies. They reanalyzed global gene expression data to correlate gene expression across diagnostic groups and than determined associations between PKC-b expression and survival. The paper is clearly written and the scientific data is very convincing regarding PKC-b expression. However the discussion/scientific impact only corroborate previous expression studies on the role of multiple critical cellular pathways such as anti-apoptosis, cell proliferation, and signalling pathways, that were found to be loosely associated with PKC-b function. A more rigorous bioinformatics study on the relationship between PKC-b expression and the other pathways would have added impact to the study. Multiple tools are available on the web for looking at co-expression and pathway analysis and should be used to strength the authors findings. Many of these tools would also add significance the pathway/association analysis, which was found missing from this study. The elevated PKC-b levels tied to prognosis is an interesting finding that should be of interest to the general readership.

#### Author's response

*We appreciate that the reviewer pointed out "Scientific quality of the work is high and should be of general interest" and "The paper is clearly written and the scientific data is very convincing". We have made revisions to address reviewer's comments. Similar to our response to the 2^nd ^reviewer's report, we modified the abstract and the discussion section to highlight the novelty of the study. We have performed additional analysis to identify genes with expressions highly correlated with PKC-β, followed by mapping them to biological pathways. These analyses confirmed our results in *Figure 6* and *Table 3. *Please see below for detailed response to each of reviewer's specific comments*.

Specific Comments:

1. Genes not represented between two different population and different array platforms or different analysis algorithms should at have identify similar pathways if not exact genes. (17 vs. 13 genes). Is this possible with your findings? Also, the authors could reanalyze this data starting from raw microarray data to see if similar algorithms derive the same answer.

#### Author's response

*The issue has been widely studied recently and it has been demonstrated that even though different gene sets were used for prognostication in cancer patients, they showed significant agreement in the outcome predictions and are probably tracking a common set of biologic phenotypes. Please see a recent publication on New England Journal of Medicine: Fan C, Oh DS, Wessels L, Weigelt B, Nuyten DS, Nobel AB, van't Veer LJ, Perou CM. (2006) Concordance among gene-expression-based predictors for breast cancer. N Engl J Med. 355(6):560-9. This topic is beyond the scope of the current study*.

2. How was the data sorted into quartiles? It appears arbitrary and not statistical as written. Would the use of supervised and unsupervised methods distinguish between the same groupings of data and would these methods validate the significance?

#### Author's response

*In our study, we applied two approaches in analyzing the correlation between PKC-β expression and patient survival. In one approach, we used the Kaplan-Meier survival analysis after grouping patients into 4 quartiles. We also applied Cox Proportional-Hazards Regression model to analyze the same dataset and reached the same conclusion. However, we only reported the Kaplan-Meier analysis results in the original manuscript. To address reviewer's concern, we added a sentence in the result section to indicate that the Kaplan-Meier analysis results were confirmed by the Cox Proportional-Hazards Regression model*.

3. Table two should be moved to supplemental data

#### Author's response

*We also considered the option of moving *Table 2* to a supplemental file. However, in the 2^nd ^reviewer's report, the reviewer pointed out the importance of analyzing probe sequences in both cDNA and Affymetrix array platforms to exclude the possibility of cross hybridization. Therefore, we feel it is necessary to show our analysis and discussion of probe sequences in *Table 2.

4. Table 3 gene list should be summarized to include significance based on comparing to background array data. The individual gene list could be moved to supplemental data.

#### Author's response

Table 3* has been modified to only include gene categories. The individual gene list has been moved into *Additional file 1*. The main text has also been modified accordingly*.

5. Figures 2, *and *5A* are not necessary*.

#### Author's response

Figure 2* and *5A* provide a direct visualization of the distribution of different PKC-β quartiles in DLBCL subtypes. We feel that it is better to describe the results using both figures and text*.

6. Figure 4 is misaligned obscuring the y axis.

#### Author's response

*We appreciate the reviewer pointed this out and it has been fixed*.

7. Graphs should be redone without color to better illustrate the data.

#### Author's response

*As we discussed in response to the 2^nd ^reviewer's report, the figures were generated using the software MedCalc (see the Method's section). We feel the readers should be able to better distinguish different survival curves that were represented by different colors*.

## Supplementary Material

Additional file 1 Genes over expressed in high PKC-beta expressing DLBCL. UniGene ID, description and classification of genes that are over expressed in the highest PKC-beta expressing (quartile 4) DLBCL patients. Click here for file
