# Supplementary material for: Retrospective analysis of protein kinase C-beta (PKC-β) expression in lymphoid malignancies and its association with survival in diffuse large B-cell lymphomas
Source: Biol Direct. 2007 Feb 21;2:8. doi: 10.1186/1745-6150-2-8 (PMC1805741; doi:10.1186/1745-6150-2-8)
Supplement: Additional file 1 — Genes over expressed in high PKC-beta expressing DLBCL. UniGene ID, description and classification of genes that are over expressed in the highest PKC-beta expressing (quartile 4) DLBCL patients. [file 1745-6150-2-8-S1.doc]

## Genes over-expressed in the highest PKC-β expressing (quartile 4) DLBCL patients

| **UniGene Cluster ID** | | **Gene name** |
| --- | --- | --- |
| ***Anti-apoptosis*** | | |
| Hs.10031 | tumor up-regulated CARD-containing antagonist of caspase nine | |
| Hs.109150 | SH3-domain binding protein 5 (BTK-associated) | |
| Hs.145279 | SET translocation (myeloid leukemia-associated) | |
| Hs.149957 | ribosomal protein S6 kinase, 90kD, polypeptide 1 | |
| Hs.152601 | UDP-glucose ceramide glucosyltransferase | |
| Hs.154210 | endothelial differentiation, sphingolipid G-protein-coupled receptor, 1 | |
| Hs.159494 | Bruton agammaglobulinemia tyrosine kinase | |
| Hs.286124 | CD24 antigen (small cell lung carcinoma cluster 4 antigen) | |
| Hs.326248 | Programmed cell death 4 (neoplastic transformation inhibitor) | |
| Hs.58831 | regulator of Fas-induced apoptosis | |
| Hs.79241 | B-cell CLL/lymphoma 2 | |
| Hs.81170 | pim-1 oncogene | |
| ***Signaling in B-cell proliferation, differentiation*** | | |
| Hs.116481 | CD72 antigen | |
| Hs.130881 | B-cell CLL/lymphoma 11A (zinc finger protein) | |
| Hs.153053 | CD37 antigen | |
| Hs.153261 | Immunoglobulin heavy constant mu | |
| Hs.153487 | signal transducing adaptor molecule (SH3 domain and ITAM motif) 1 | |
| Hs.167746 | B-cell linker | |
| Hs.169832 | zinc finger protein 42 (myeloid-specific retinoic acid- responsive) | |
| Hs.1706 | interferon-stimulated transcription factor 3, gamma (48kD) | |
| Hs.191958 | Immunoglobulin superfamily receptor translocation associated 2 | |
| Hs.192861 | Spi-B transcription factor (Spi-1/PU.1 related) | |
| Hs.194976 | SH2 domain-containing phosphatase anchor protein 1 | |
| Hs.327 | interleukin 10 receptor, alpha | |
| Hs.54452 | zinc finger protein, subfamily 1A, 1 (Ikaros) | |
| Hs.54460 | small inducible cytokine subfamily A (Cys-Cys), member 11 (eotaxin) | |
| Hs.58685 | CD5 antigen (p56-62) | |
| Hs.64310 | interleukin 11 receptor, alpha | |
| Hs.73958 | recombination activating gene 1 | |
| Hs.74101 | spleen tyrosine kinase | |
| Hs.82212 | CD53 antigen | |
| Hs.87205 | lymphocyte antigen 64 homolog, radioprotective 105kD (mouse) | |
| Hs.89714 | small inducible cytokine subfamily B (Cys-X-Cys), member 5 (epithelial-derived neutrophil-activating peptide 78) | |
| Hs.901 | CD48 antigen (B-cell membrane protein) | |
| ***Cancer development*** | | |
| Hs.118722 | fucosyltransferase 8 (alpha (1,6) fucosyltransferase) | |
| Hs.1369 | decay accelerating factor for complement (CD55, Cromer blood group system) | |
| Hs.157441 | spleen focus forming virus (SFFV) proviral integration oncogene spi1 | |
| Hs.1741 | integrin, beta 7 | |
| Hs.238730 | NORE1 protein | |
| Hs.35384 | ring finger protein 1 | |
| Hs.356386 | RAB7, member RAS oncogene family | |
| Hs.73722 | APEX nuclease (multifunctional DNA repair enzyme) | |
| Hs.78781 | vascular endothelial growth factor B | |
| Hs.79440 | IGF-II mRNA-binding protein 3 | |
| Hs.80887 | v-yes-1 Yamaguchi sarcoma viral related oncogene homolog | |
| Hs.81256 | S100 calcium binding protein A4 (calcium protein, calvasculin, metastasin, murine placental homolog) | |
| Hs.81687 | non-metastatic cells 3, protein expressed in | |
| Hs.82028 | Transforming growth factor, beta receptor II (70-80kD) | |
| Hs.846 | interleukin 8 receptor, beta | |
| Hs.85701 | phosphoinositide-3-kinase, catalytic, alpha polypeptide | |
| Hs.9235 | non-metastatic cells 4, protein expressed in | |
| Hs.99491 | RAS guanyl releasing protein 2 (calcium and DAG-regulated) | |
| ***Cell proliferation/cell-cycle control*** | | |
| Hs.109752 | putative c-Myc-responsive | |
| Hs.165843 | casein kinase 2, beta polypeptide | |
| Hs.172690 | diacylglycerol kinase, alpha (80kD) | |
| Hs.7019 | signal-induced proliferation-associated gene 1 | |
| Hs.850 | IMP (inosine monophosphate) dehydrogenase 1 | |
| Hs.138860 | Rho GTPase activating protein 1 | |
| Hs.153640 | cytokine-inducible kinase | |
| Hs.153704 | NIMA (never in mitosis gene a)-related kinase 2 | |
| Hs.155530 | interferon, gamma-inducible protein 16 | |
| Hs.1592 | CDC16 cell division cycle 16 homolog (S. cerevisiae) | |
| Hs.184167 | splicing factor, arginine/serine-rich 7 (35kD) | |
| Hs.2331 | E2F transcription factor 5, p130-binding | |
| Hs.347349 | cell cycle progression 2 protein | |
| Hs.57101 | MCM2 minichromosome maintenance deficient 2, mitotin (S. cerevisiae) | |
| Hs.75586 | cyclin D2 | |
| Hs.77171 | MCM5 minichromosome maintenance deficient 5, cell division cycle 46 (S. cerevisiae) | |
| Hs.77313 | cyclin-dependent kinase (CDC2-like) 10 | |
| Hs.77613 | ataxia telangiectasia and Rad3 related | |
| Hs.9700 | cyclin E1 | |
| ***DNA repair*** | | |
| Hs.108327 | damage-specific DNA binding protein 1 (127kD) | |
| Hs.109526 | zinc finger protein 198 | |
| Hs.18895 | tousled-like kinase 1 | |
| Hs.192803 | xeroderma pigmentosum, complementation group A | |
| Hs.197345 | thyroid autoantigen 70kD (Ku antigen) | |
| Hs.35947 | methyl-CpG binding domain protein 4 | |
| Hs.48576 | excision repair cross-complementing rodent repair deficiency, complementation group 5 (xeroderma pigmentosum, complementation group G (Cockayne syndrome)) | |
| Hs.59544 | excision repair cross-complementing rodent repair deficiency, complementation group 1 (includes overlapping antisense sequence) | |
| Hs.75462 | BTG family, member 2 | |
| Hs.79396 | N-methylpurine-DNA glycosylase | |
| Hs.84318 | replication protein A1 (70kD) | |
| Hs.99987 | excision repair cross-complementing rodent repair deficiency, complementation group 2 (xeroderma pigmentosum D) | |
| ***DNA, RNA, protein synthesis*** | | |
| Hs.111611 | ribosomal protein L27 | |
| Hs.155202 | Transcription elongation factor B (SIII), polypeptide 3 (110kD, elongin A) | |
| Hs.169793 | ribosomal protein L32 | |
| Hs.275865 | ribosomal protein S18 | |
| Hs.326249 | ribosomal protein L22 | |
| Hs.351937 | ribosomal protein, large P2 | |
| Hs.356473 | activated RNA polymerase II transcription cofactor 4 | |
| Hs.539 | ribosomal protein S29 | |
| Hs.5398 | guanine monophosphate synthetase | |
| Hs.75782 | general transcription factor IIIC, polypeptide 2 (beta subunit, 110kD) | |
| Hs.76194 | ribosomal protein S5 | |
| Hs.77039 | ribosomal protein S3A | |
